# Improving the Magnetic Properties of Non-Oriented Electrical Steels by Secondary Recrystallization Using Dynamic Heating Conditions

**DOI:** 10.3390/ma12121914

**Published:** 2019-06-13

**Authors:** Ivan Petryshynets, František Kováč, Branislav Petrov, Ladislav Falat, Viktor Puchý

**Affiliations:** 1 Institute of Materials Research, Slovak Academy of Sciences, Watsonova 47, 04001 Košice, Slovakia; fkovac@saske.sk (F.K.); branislav.petrov@embraco.com (B.P.); lfalat@saske.sk (L.F.); vpuchy@saske.sk (V.P.); 2 Faculty of Materials, Metallurgy and Recycling, Technical University of Košice, Letná 9, 04200 Košice, Slovakia; 3 Embraco Slovakia, s.r.o., Odorínska cesta 2, 052 01 Spišská Nová Ves, Slovakia

**Keywords:** electrical steel, crystallographic texture, magnetic losses, efficiency of electrical motor

## Abstract

In the present work, we have used unconventional short-term secondary recrystallization heat treatment employing extraordinary high heating rate to develop coarse-grained microstructure with enhanced intensity of rotating cube texture {100}<011> in semi-finish vacuum degassed non-oriented electrical steels. The soft magnetic properties were improved through the increase of grains size with favourable cube crystallographic orientation. The appropriate final textural state of the treated experimental steels was achieved by strain-induced grain boundary migration mechanism, activated by gradient of accumulated stored deformation energy between neighbouring grains after the application of soft cold work, combined with steep temperature gradient during subsequent heat treatment under dynamic heating conditions. The materials in our experimentally prepared material states were mounted on the stator and rotor segments of electrical motors and examined for their efficiency in real operational conditions. Moreover, conventionally long-term heat treated materials, prepared in industrial conditions, were also tested for reference. The results show that the electrical motor containing the segments treated by our innovative approach, exhibits more than 1.2% higher efficiency, compared to the motor containing conventionally heat treated materials. The obtained efficiency enhancement can be directly related to the improved microstructural and textural characteristics of our unconventionally heat treated materials, specifically the homogenous coarse grained microstructure and the high intensity of cube and Goss crystallographic texture.

## 1. Introduction

The non-oriented (NO) electrical steels belong to the group of soft magnetic materials. They represent important materials used for core laminations in the majority of electrical devices, and they are contributing to the efficiency improvement of the equipment [1]. That is why the NO steels have to possess appropriate magnetic properties such as high magnetic induction, high magnetic permeability, low coercive fields, and low core losses in all plane directions [2,3]. Because of magnetic properties isotropy, these steels are typically used in various electromechanical rotating machines, e.g., in electrical motors, generators, and actuator which transform electrical energy to mechanical and vice versa [4]. It is well-known that magnetic properties of NO steels strongly depend on several different factors, mainly the chemical composition (especially, the silicon content), microstructural state (i.e., grain size and grains matrix distribution), crystallographic texture (i.e., rotated cube and Goss texture component) and other factors which also include the strip thickness, impurities, isolation, mechanical stress, sheet thickness, etc. In order to improve the magnetic permeability and reduce total power loss, the coarse-grained microstructure with <100>//ND texture (θ-fibre), which comprises the cube, rotated cube as well as all orientations with the {001} planes parallel to the sheet plane, is desired since it contains the highest number of the easy magnetization axes <001> in the sheet plane. The <111>//ND (γ- fibre) texture has the hard magnetization axes <111> in the sheet plane, and thus needs to be suppressed in the final steel sheets [5,6,7,8,9].

Non-oriented electrical steels are divided into two groups: fully-processed (i.e., fully-finished) and semi-processed (i.e., semi-finished) grade. The fully-finished grade is a final product of conventionally processed NO steel. It has the microstructure, magneto-crystalline texture, and specific magnetic properties which have been adjusted through hot band annealing, cold rolling and final annealing of thin steel strip. This material can be readily used for the final assembly by the equipment manufacturer [10,11]. 

In contrast, semi-processed electrical steels are finished to their final thickness by the steel producer and are then subjected to the cutting, stacking and final assembly by the customers. In order to eliminate any undesired effects of such operations on final magnetic properties of NO steels, their final annealing is required. After cold rolling, semi-processed steel coils are generally temper rolled (i.e., skin passed) and then they are subjected to final recrystallization annealing to improve their punchability, facilitate strain-induced grain growth during the annealing at final producers of electromechanical machines. It has been estimated that the temper rolling introduces a soft deformation into the material, which accounts for 5–10% reduction in its thickness [12,13,14]. Thus, the semi-finished steels possess some extent of stored deformation energy, accumulated in dislocation structures, which can provide a sufficient driving force for a selective grain growth process, also known as strain-induced grain boundary migration phenomenon [15]. It is generally accepted, that the stored deformation energy is proportional to the amount of slip activity, which in polycrystalline materials depends on grain orientation plane, according to the sequence E_{1 1 1}_ > E_{1 1 2}_ > E_{1 0 0}_ [16,17], where {hkl} represents the plane parallel to the rolling plane in Miller indices notations. The stored energy of cold work is therefore supposed to change from grain to grain according to the local crystallographic orientation as a function of applied stress. This means that the small rolling strains induce a gradient of local stresses between neighbouring grains, randomly distributed through the sheet thickness. Depending on their crystallographic orientations, the phenomenon of strain-induced grain migration can be used to increase the grains size during the second annealing (i.e., secondary recrystallization) treatment [18,19].

In industrial conditions, the final heat treatment of semi-finished NO electrical steels is carried out according to EN 10 341 standard [20]. In this case, the conventional long-term annealing process of steel laminations is used for the grain size increase by the mechanism of deformation-induced grain growth and the elimination of residual punching stresses, thereby eliminating all the deleterious effects affecting final magnetic properties. However, the main disadvantage of such conventional treatment comes from a limitation of the heating rate during annealing that leads to early recovery processes in the temper rolled NO steel. Consequently, such a premature onset of the recovery processes lowers the driving force for deformation-induced grain boundary motion before achieving the optimal annealing temperature. Moreover, the whole conventional process cycle: heating, annealing and cooling lasts more than 10 h.

One important type of electromechanical machines that convert electric energy into mechanical energy is represented by electric motors. Despite differences in size and type, all electric motors in principle work the same way: an electric current flowing through a wire coil in a magnetic field creates a force that rotates the coil, thus creating torque. The main core materials which provide the magnetic flux-carrying member in most electric motors are non-oriented electrical steels. Because of this reason, the final magnetic properties improving these materials allow for an increase of the electrical motor efficiency. In order to understand how the heat treatment conditions examined in this work influence the working characteristics of rotating machines, the operational testing of a electrical motor was carried out. It is important to say that four different kinds of losses occur in a motor: electrical losses, magnetic losses, mechanical losses and stray (i.e., eddy current) losses [21]. These losses can be reduced by using quality materials, as well by optimizing the design. The magnetic losses occurring in steel laminations of stator and rotor segments can be lowered via improvement of their chemical composition, microstructure, substructure, and texture parameters.

Our previous works [22,23,24,25] were focused mostly on the investigation of strain-induced selective grain growth mechanism, depending on the value of accumulated deformation achieved by the temper rolling processes. It has been presented that in NO silicon steels subjected to soft cold rolling deformation (i.e., 2-6% thickness reduction), columnar or coarsened-grains matrix with randomly distributed grains, showing significant intensity of rotated cube crystallographic orientation, could only be achieved at very high heating rates. Furthermore, the obtained experimental findings indicated that the annealing temperature has to be higher than 900 °C. After such dynamic heat treatments, the achieved microstructure and texture of the treated materials were found to have a clearly positive affect on their final magnetic properties, namely the core losses and coercivity. Even more, the proposed approach allowed not only for an improvement of the final magnetic parameters of NO steels but it also enabled a reduction of the final production costs for end customers. 

In contrast to the scientific works [26,27,28], addressing the use of unconventional cold rolling schemes (e.g., with different rolling angles to the rolling direction) and subsequent conventional heat treatment with low heating rate, we have proposed a combination of a conventional temper rolling process for the steel after primary recrystallization with a subsequent final, unconventional second annealing treatment at dynamic heating conditions, i.e., using extraordinary high heating rate. On the other hand, other research studies [29,30] have clearly indicated a beneficial effect of rapid annealing on the increase of average grain size and significant improvement of crystallographic texture of electrical steel after strip-casting and cold rolling process.

In the present study, rotor and stator segments, manufactured by shear cutting of semi-finished steel, were subjected to the second annealing treatment in our laboratory dynamic heating conditions. The main aim of present investigation was to compare and discuss the results obtained from operational torque load tests of electrical motors assembled from NO steel laminations, individually heat treated either in laboratory or industrial heat treatment conditions.

## 2. Materials and Methods 

**Figure 1:**
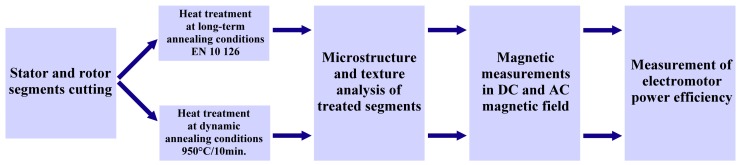
Scheme of experimental procedures which were used for investigated steel.

The material investigated in this work was a commercially available semi-finished non-oriented electrical steel of M450-50A grade, in the form of sheets with 0.5 mm in thickness and following chemical composition in wt.%: Fe = 97.95%, C = 0.006%, Si = 1.48%, Mn = 0.25%, P = 0.040%, Al = 0.18%, other elements ~0.094%. Schematic overview of experimental procedures, which were involved in experimental investigation of the studied steel, is displayed in Figure 1.

**Figure 2:**
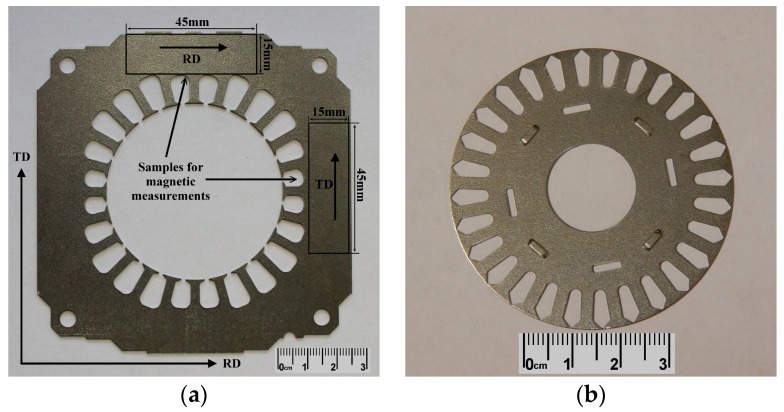
Experimental samples in form of stator (**a**) and rotor (**b**) segments of electric motor.

The experimental samples were in form of stator and rotor segments (see Figure 2). These segments were prepared by shear cutting in industrial conditions, in accordance with common electro-motors manufacturing technology. More than 80 experimental segments were heat treated in laboratory conditions and then used as core material of rotor and stator in electrical motor. The final performance parameters of experimentally prepared rotating machine (i.e., by using laboratory heat treated segments) were compared with those of the electrical motor assembled, according to conventional industrial technology (i.e. by using industrially heat treated segments).

**Figure 3:**
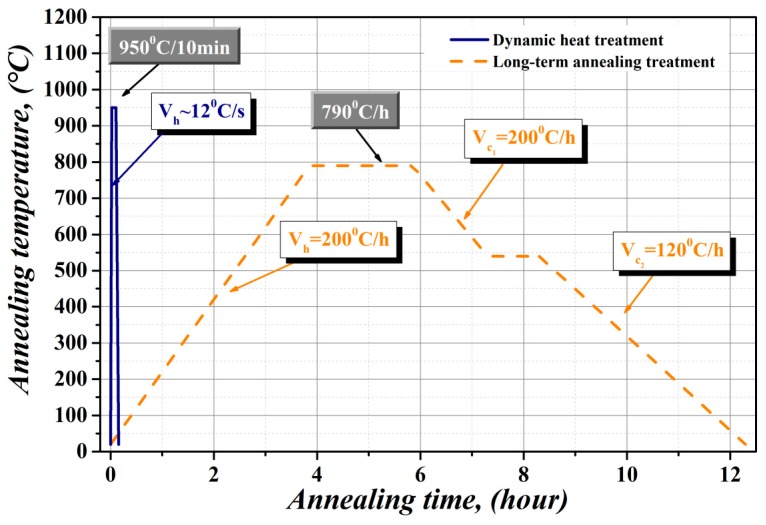
Schematic representation of individual heat treatment processes of experimental material: solid blue line—unconventional dynamic heat treatment of stator and rotor laminations and dashed yellow line—conventional long-term annealing treatment at industrial conditions.

The heat treatments of experimental segments were individually carried out at either unconventional dynamic heating or conventional long-term annealing conditions, according to the regimes schematically shown in Figure 3. The dynamic heat treatment of experimental segments was performed in laboratory conditions using electric resistance furnace Nabertherm RS 120/1000/13 (Nabertherm GmbH, Lilienthal, Germany). The stator and rotor laminations were heated up to 950 °C at a heating rate of about 12 °C/s (see the solid blue line in Figure 3). Then, the segments were kept at the annealing temperature for 10 min. The annealing atmosphere was pure hydrogen, d.p. (dew point) ~25 °C. Due to dimensional limitations of the work space at the laboratory furnace used, our laboratory heat treatment of large number of electro-motor core segments was carried out sequentially, i.e., a series of maximum five segments on a special in-house made sample holder was heat treated per single annealing process. This procedure was repeated until full completion the heat treatment for all the experimental segments.

The long-term annealing treatment was carried out at an industrial line of conventional heat treatment according to the EN 10 341 standard and it is schematically presented by the dashed yellow line in Figure 3. The duration of conventional industrial heat treatment was about 12.5 h. It is important to note that the final phase of cooling process included also the treatment at 540 °C/1 h in a special atmosphere, which provided the formation of so-called “surface blue” isolation layer on the base of complex iron (II, III) oxide Fe_3_O_4_, i.e., FeO·Fe_2_O_3_. This layer (typical 0.5–1.5 μm) is responsible for the isolation between each segment in stator and rotor of electrical motors and thus it reduces eddy-current losses. In order to achieve required isolation layer on the surface of rotor and stator segments heat treated in laboratory conditions, they were additionally processed within the final separated box of industrial heat treatment line.

The selected representative samples were used for the microstructure and texture analyses. The texture analysis was carried out by means of electron back-scattered diffraction (EBSD) method in the normal direction plane for each sample of 25 mm × 10 mm in size. The scanning electron microscope (SEM) JEOL JSM 7000F FEG (Jeol Ltd., Tokyo, Japan) with the EBSD detector Nordlys-I (HKL technology A/S, Hobro, Denmark) were used to perform the texture analysis. The obtained EBSD data were processed by the CHANNEL-5, HKL software package (Service pack 7).

The magnetic properties of the segments, heat treated according to both individual annealing schemes, were measured on the samples with planar dimensions 45 mm × 15 mm. These samples were prepared by electrical discharge machining using spark erosion machine EIR-EMO 2N (Emotek s.r.o., Nové Mesto nad Váhom, Slovak Republic). In order to evaluate the magnetic properties of the heat treated segments in two main directions, these samples were cut from the segments along the rolling direction (RD) and transverse direction (TD), see Figure 2. The magnetic measurements in direct current (DC) and alternating current (AC) magnetic field conditions were carried out using magnetic measuring instrument Brockhaus MPG 100D (Dr. Brockhaus Messtechnik GmbH & Co. KG, Lüdenscheid, Germany). The AC hysteresis loop measurements were performed at a frequency of 50 Hz.

The measurements of electrical motors efficiency, which were constructed from the segments heat treated in either short-term (laboratory) dynamic heating or long-term (industrial) static annealing conditions, were carried out by operational torque load testing of electrical rotating machines at the electric motor test station on the manufacturer workplace. For the efficiency evaluation, a so-called direct method was used, which is generally considered to be very accurate. The measurement of the efficiency of electric motors was made directly using the equation:

(1) $$\mathrm{Efficiency}\;\%=\frac{\mathrm{Mechanical}\;\mathrm{Output}\;\mathrm{Power}\;\cdot 100\%}{\mathrm{Electrical}\;\mathrm{Input}\;\mathrm{Power}},$$

Thus, it was required to measure both the mechanical output power and the electrical input power. The electric input power was measured with satisfactorily good accuracy. The mechanical power was given by the equation:

Mechanical Power = Torque · Angular Speed(2)

The speed measurement is a relatively simple procedure which can provide the achievement of quite accurate results (±1 rpm). The torque measurement was carried out by dynamometer model DINA06TRO07 (Kropy industrial Ltd., Joinville, Brazil), which had the possibility of creating a controllable variable load, fitted with an accurate torque transducer.

## 3. Results and Discussion

### 3.1. Microstructure

**Figure 4:**
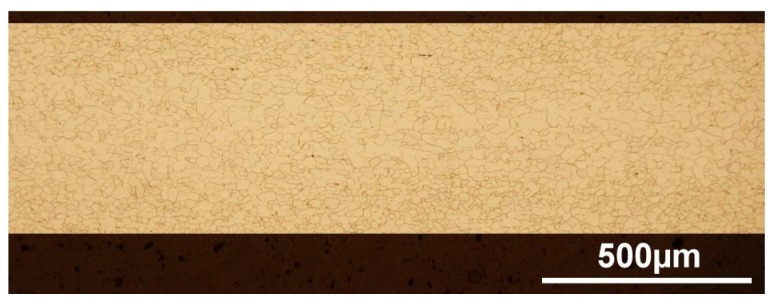
The initial microstructure of stator segment obtained from semi-finished non-oriented steel before heat treatment.

Representative samples from the electric motor segments, heat treated using the two different heat treatment conditions, were metalographically prepared for microscopic analysis of their microstructural state using light optical microscope OLYMPUS GX71 (OLYMPUS Europa Holding GmbH, Hamburg, Germany). The evaluation of main microstructural parameters was carried out through the sheet plane cross-section parallel to the rolling direction. The initial microstructure of stator and rotor segments produced of semi-finished non-oriented steel before the heat treatment is shown in Figure 4. It can be seen that the experimental steel in its initial material state is characterised by quite fine-grained homogenous microstructure with an average grain size of 17 μm ± 3 μm. 

**Figure 5:**
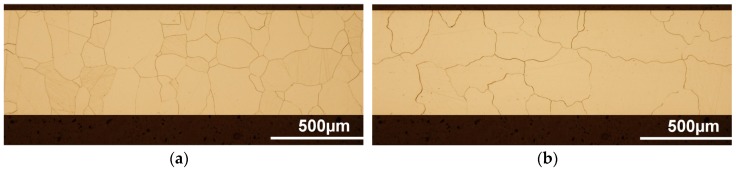
The microstructure of experimental stator segments after: conventional long-term heat treatment (**a**) and unconventional short-term dynamic heat treatment (**b**).

The grain matrix evolutions of investigated segments subjected to the secondary recrystallization annealing in conventional long-term and unconventional dynamic annealing conditions are shown in Figure 5a,b, respectively. It can be clearly seen that both samples have the monophasic ferrite coarse-grained microstructure, which comply with requirements for the final magnetic properties of rotating equipment core material. In the case of the segments treated according to the EN 10 341 standard, the cross-section microstructure is composed of inhomogeneous alternation of grains with columnar and/or equiaxial symmetric structure. The light optical microscopic analysis shows that the conventionally heat treated segments are characterized by the average grain size of 120 μm ± 10 μm, see Figure 5a. The microstructural features of laminations, which were heat treated at dynamic conditions at 950 °C during 10 min, is presented in Figure 5b. The light optical image demonstrates that the unconventionally heat treated steel is characterised by coarse grains matrix with a mean grain size of 250 μm ± 10 μm. 

Evidently, most of the grains have a uniaxial shape with warped boundaries and are mutually contiguous to each other in the middle part of the cross-section. Such grains morphology reasonably leads to the conclusion that they grow from the sheet sub-surface region to its central part. It should be noted that in the case of the samples heat treated according to the industrial scheme, a similar tendency of the growth of the grains was not so obvious. On the contrary, the microstructure in Figure 5a contains a lot of grains, with their size ranging from 40 μm up to 200 μm, located mostly in central part of the samples. This arrangement of grains may be due to the effect of a simultaneous growth of large number of grains through the thickness of the sample. It is apparent, that the observed microstructural differences of individual experimental segments can be directly related to the used conditions of performed secondary heat treatments, which were differing from each other by their heating rate, annealing temperature and holding time. It is well-known that secondary recrystallization [31] leads to further migration of grain boundaries through the primary recrystallized structure, thereby producing a structure containing a small number of enlarged grains. The driving force for the growth of new grains is provided by the removal of the storage energy associated with a plastically deformed material state. In principle, a gradient of any intensive thermodynamic variable offers a source of such a driving force. In the case of the semi-finished non-oriented steel material obtained after the temper rolling process, with a sheet thickness reduction ranging from 3% to 10%, the soft cold work deformation energy is stored within dislocation structures. The plastic anisotropy of grains in a polycrystalline microstructure results in a stored energy difference across grain boundaries, which in turn can provide a driving force sufficient for so-called strain-induced grain boundary migration (SIBM). Further annealing of semi-processed steel laminations may result in grain growth, in which the smaller primary recrystallized grains are eliminated, the larger grains grow, and the grain boundaries assume a lower energy configuration. The microstructural analyses of the heat treated segments have shown that the strain-induced grain boundary migration mechanism enabled to achieve the microstructures with a smaller number of enlarged grains which had the desired effect on their magnetic properties. However, the difference in morphology of finally obtained microstructures indicate that in the case of secondary grain growth obtained in our laboratory dynamic heating conditions, the microstructure evolution is affected not only by the stored deformation energy, but also by the driving force related to the heat transfer phenomena during the thermal processing, see Figure 5a,b. Special attention is given to the temperature distribution during the heating process. In case of the long-term annealing process, the heating rate is very slow, and the heating of the segments is homogeneous throughout the segments cross-section. These heating conditions enable the progress of recovery processes at lower temperatures and thus decreased the driving force associated with deformation storage energy, which is responsible for the SIBM. Moreover, long-term annealing gives an opportunity to develop a large number of primary recrystallized grains randomly distributed throughout the steel sheet thickness. As a result, the microstructure with the smaller average grain size was obtained after the conventional heat treatment, see Figure 5a. On the contrary, the dynamic heat treatment procedure was characterised by the high heating rate. Sidor et al. [32] showed that rapid heating is responsible for the occurrence of temperature gradient along the normal of the sheet thickness. The strong temperature gradient is an additional major driving force, which in combination with the storage deformation energy within the short annealing time, results in an intensive growth of a smaller number of primary recrystallized grains, which mostly start to grow from the sheet surface and then continue in growth towards the sheet central part. It is evident from Figure 5b that the similar grain growth was also achieved for the segment heat treated in dynamic heating conditions. However, the obtained microstructure (Figure 5b) is characterised not only by a lower number of grains with much larger average size than in microstructure of the segments obtained after long-term annealing (Figure 5a), but also by columnar and/or uniaxial grains related to their specific growth features.

### 3.2. Texture

**Figure 6:**
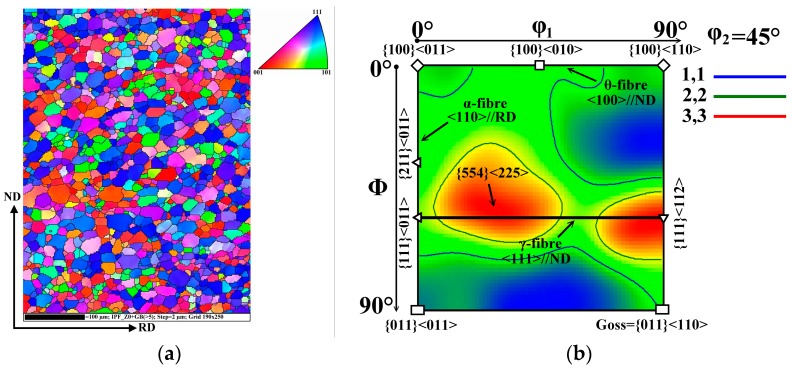
Characterization of the textural state of experimental steel in initial semi-finished state: IPF map (**a**) and ODF section taken at ϕ_2_ = 45° (**b**). The key for the identification of crystallographic orientation of grain is located in the upper right corner of the IPF map.

The crystallographic orientation or texture is an important parameter describing the magnetic properties of NO electrical steels. In order to characterize the texture evolution of the investigated segments of semi-finished NO steel in dependence of heat treatment conditions, specific EBSD analyses were performed. The common texture of experimental steel in its initial material state is shown in Figure 6. The crystallographic orientation of primary recrystallized grains matrix is represented by an inverse pole figure (IPF) map and the orientation distribution function (ODF) in Figure 6a,b, respectively. The EBSD data show that the fine-grained microstructure through the sheet thickness is characterised mostly by grains with deformation texture {111}<uvw> (blue grains) and rotated cube {001}<110> (red grains) texture, see Figure 6a. The most relevant texture components of this sample are α-, γ-, and θ- fibres represented by ODF in Figure 6b. It can be clearly seen that the semi-finished state is characterised by the dominance of γ-fibre, which represents the deformation texture <111>//ND with two characteristic maxima {111}<112> and {554}<225>. This crystallographic texture component deteriorates the magnetic properties of silicon steel. However, the θ- fibre of ODF section (Figure 6b) as well as the IPF map (Figure 6a) clearly demonstrate the presence of weak peaks of rotated cube {001}<110> grains, which are characterised by <100> directions and are responsible for easy magnetization of Fe-single crystal. 

**Figure 7:**
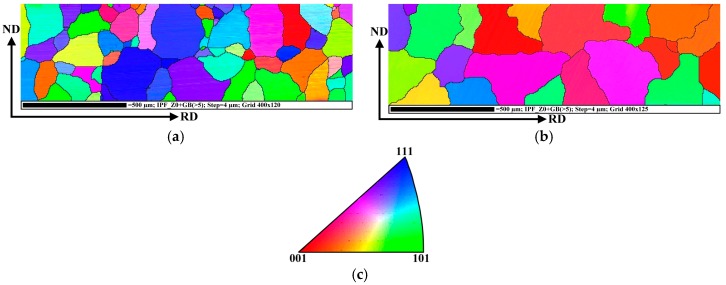
IPF map of the segments treated in long-term (**a**) and dynamic (**b**) annealing conditions. The key for the identification of crystallographic orientation of grains (**c**). Here, the red colour represents the cube crystallographic orientation, the green colour—Goss crystallographic orientation and blue colour—the deformation texture.

The IPF maps obtained on the segments treated in long-term and dynamic annealing conditions are presented in Figure 7a,b, respectively. It can be seen that the secondary recrystallization generally improved the crystallographic textures and the maximum intensities have been considerably modified, compared to the initial material state. 

It is clearly visible that the recorded EBSD IPF maps (Figure 7) show the same microstructural characteristics as light optical imagines in Figure 5. 

**Figure 8:**
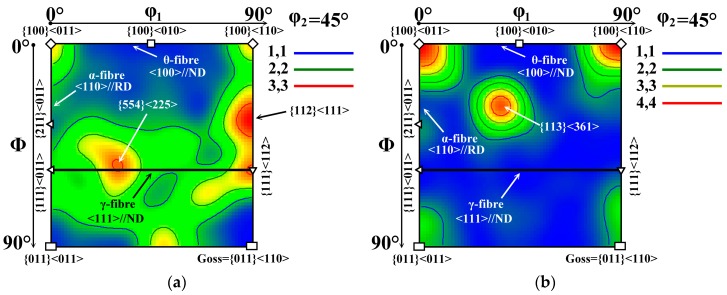
ODF sections taken at ϕ_2_ = 45° representing the through-thickness textures evolved after heat treatment in long-term (**a**) and dynamic (**b**) heat treatment conditions.

A comparison of the two IPF maps in Figure 7 clearly reveals that the segments annealed in dynamic conditions not only have a much better grains matrix than those treated in static conditions but they are also characterised by a high intensity of grains with appropriate {100} easy magnetisation axis parallel to the normal direction to the sheet plane. In this case, the grains with rotated cube component cover about 50% of analysed cross-section microstructure (see Figure 7b). The IPF map in Figure 7a shows that secondary recrystallized grains of the segments, which were heat treated according to the EN 10 341 standard, are characterised mostly by the deformation texture {111}//ND and weak intensity of rotated cube as well as Goss texture component. The resulting ODFs for the coloured IPF maps presented in Figure 7a,b are shown in Figure 8a,b, respectively. Here, the evolution of crystallographic texture of the heat treated segments is clearly characterized in terms of main fibre components. It is important to note that these results represent the totally different arrangements of the texture components, compared to those observed in the initial semi-finished material state (Figure 6). In the case of the sample in Figure 8a, it is visible that the texture is still characterised by domination of γ-fibre with high intensity of {111}<112> and {554}<225> components.

On the other hand, the rotated cube texture, represented by θ-fibre, has partially increased in the area of {100}<011> and {100}<110> components. The texture of the segments heat treated in dynamic heating conditions is represented by ODF section in Figure 8b. A quick visual inspection shows that compared to the primary recrystallized state (Figure 6b), the three common texture fibres are totally changed after the secondary recrystallization. It is important to note that γ-fibre in this case was totally reduced in comparison with initial state where the deformation texture had the maximum intensity. Moreover, the θ-fibre was significantly improved. It is evident that the maximum intensity was achieved for the grains with rotated cube components {100}<011> and {100}<110>. However, there is also a high intensity of {113}<361> component. The {113}<361> orientation belongs to the {11h}<12$\frac{1}{2}$> fibre [33] and is commonly found in low carbon steels after cold rolling and annealing. Therefore, it is believed that the {113}<361> component might have resulted from the rotated cube, as it has also been pointed out in [34].

The EBSD measurements of the experimental segments clearly showed that secondary recrystallization conditions allow for the control of not just the evolution of their microstructure, but also have a crucial effect on their resulting crystallographic texture. It has been presented that the high heating rate by dynamic heat treatment promotes the increase of rotated cube texture component despite of high intensity of deformation texture <111>//ND, which dominated in the primary recrystallized matrix. It can be concluded that the strain-induced grain boundary migration mechanism in combination with heat flow gradient induce the mostly selective grain growth, which is characterised by the crystal lattice with ease magnetisation axis <100> parallel to the ND of steel plane. The authors [18,30] concluded that the stored deformation energy in polycrystalline materials, which were subjected to cold deformation is distributed heterogeneously between the grains and depends on their crystallographic orientation, at least on orientations families defined by the crystallographic plane lying parallel to the sheet plane. In vacuum degassed ferrite steels, the stored deformation energy can be categorized as follows: E_{1 1 1}_ > E_{1 1 2}_ > E_{1 0 0}_. As one can see, the stored energy increase from the grains with {001}<110> to the grains with {111}<110>crystallographic orientation. It can be supposed that between the neighbouring grains with various crystallographic orientations, a creation of some small gradient of stored deformation energy is related to the lattice defects, such as dislocations, which were induced by the applied mechanical stress. The gradient of stored energy represents a driving force for strain-induced grain boundary migration mechanism and enables promoting the migration of grains with less internal energy [35]. Based on all mentioned information, it can be supposed that semi-finished electrical steels are characterised by a randomly distributed stored deformation energy gradient between the primary recrystallized grains. The present stresses disparity on the grains boundaries in combination with steep temperature gradient during the secondary dynamic annealing allow to obtain the growth of a small number of grains with appropriate rotated cube crystallographic orientation. This statement is directly supported by the results obtained in present investigation showing adequate correlation between microstructure (see Figure 5b) and texture (see Figure 7b and Figure 8b) characteristics. From comparison of the microstructural and textural states of the segments heat treated in long-term and dynamic annealing conditions, it is possible to conclude that the temperature gradient creates the conditions in temper rolled polycrystalline materials for the evolution of secondary grains matrix with preferential rotated cube crystallographic orientation.

### 3.3. Magnetic Measurements

**Figure 9:**
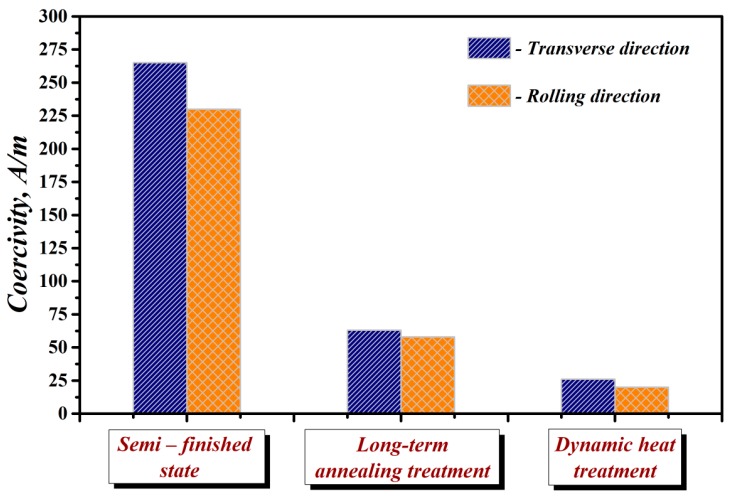
Dependence of the heat treated segments coercivity on the annealing conditions and radial direction.

A precise estimation of the relationship between the microstructure and texture in the investigated steel can be obtained through the measurements of final magnetic properties. It is well-known that the texture of NO electrical steels has a profound effect on the magnetic properties, e.g., total power losses, permeability, coercivity, induction, etc. of the core laminations [36,37]. Since these magnetic materials are strongly textured and the texture is a crucial source of anisotropy, the magnetic properties are conventionally measured at various angles with respect to the rolling direction. In order to evaluate the magnetic anisotropy of the investigated steel, the testing samples were prepared in the rolling direction (RD) and the transverse (TD) direction, as it was shown in Figure 2a. The samples obtained from the initial semi-finished material state as well as from the segments annealed in different conditions were subjected to the measurements in the DC and AC magnetic field. The Figure 9 shows a dependence of the measured DC coercivity of the investigated samples on the radial directions. Representative magnetic measurements were carried out in order to differentiate the samples performance after different heat treatment procedures. The measurements clearly indicated some anisotropy and clear differences between magnetic properties of the segments heat treated under laboratory (dynamic) and industrial (long-term) conditions.

**Figure 10:**
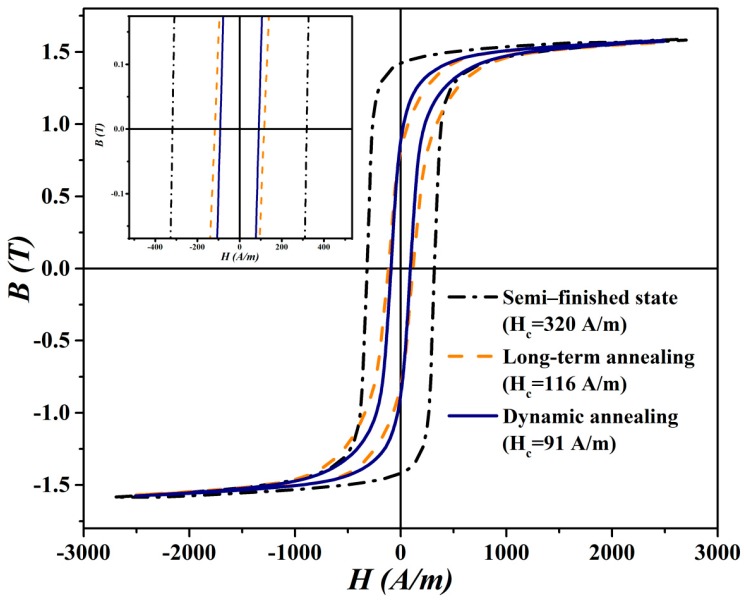
The measured B-H loops at 50Hz with peak flux densities at 1.7 T.

From Figure 9, it is evident that coercivity is much higher for the samples in an initial semi-finished material state than for the heat treated ones. Moreover, it should be noted that the initial steel is characterised by its pronounced anisotropy. In this case, the coercivity values in the RD and TD directions were determined to be 230 A/m and 265 A/m, respectively. On the other hand, the segments subjected to both performed heat treatments show a significant reduction in their coercivity values, compared to those without heat treatment. The obtained coercivity values in the RD for the samples heat treated in long-term and dynamic conditions are 58 A/m and 20 A/m, respectively. The obtained coercivity values in the TD for the samples heat treated in long-term and dynamic conditions are 63 A/m and 26 A/m, respectively. This indicates that the annealing processes which are responsible for the evolution of microstructural and textural characteristics of the studied steel clearly improve its magnetic properties as well. Moreover, it has been demonstrated that the regime of annealing may also have a significant influence on the value of final coercivity. The segments after the heat treatments are characterized by very weak anisotropy which is acceptable for core materials of electric-rotated machines. Comparison of the samples heat treated in industrial and laboratory conditions clearly show that the lowest coercivity values were obtained for the segments heat treated in dynamic laboratory conditions. This result perfectly corresponds with an observed evolution of microstructural and textural characteristics of the investigated materials. The evolution of core losses of experimental samples prepared in the RD was carried out also in AC magnetic field with a frequency of 50 Hz. The results of these measurements are presented by typical B-H loops in Figure 10. It is well-known that the area enclosed by the B-H loop represents the core losses. Here, the maximum value of core losses was obtained for the segments of the investigated steel before heat treatment (i.e., in initial semi-finished material state). The AC magnetic measurements results are also presented in Table 1. In the case of the heat treated samples, it is obvious that the segments annealed in dynamic laboratory conditions have much lower core loss than the segments heat treated in industrial conditions according to EN 10 341 standard. 

It can be clearly seen that these measurements (Figure 10, Table 1) perfectly correspond with the results which were obtained in the DC magnetic field (Figure 9). Moreover, the data of B-H hysteresis loops show that the steel segments heat treated in dynamic conditions show more than a 20% decrease of core losses in comparison with the annealing process used in industrial conditions (see Table 1).

### 3.4. Measurement of Efficiency

**Figure 11:**
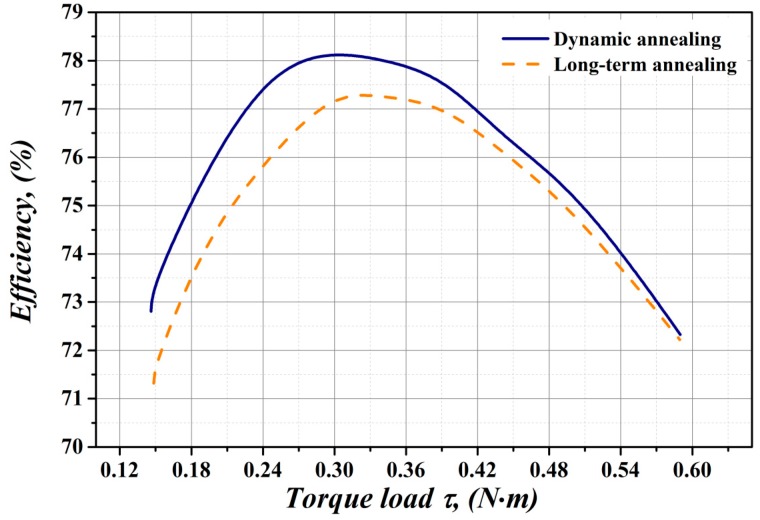
Efficiency dependence of electrical motors constructed from the segments heat treated in dynamic (solid blue line) and long-term (dashed orange line) conditions on the torque load.

In the present work, the tested electric motors were assembled from rotor and stator segments heat treated in either conventional long-term or unconventional short-term dynamic annealing conditions. The results of the efficiency measurements within the working torque load range of the experimental motors are presented in Figure 11. 

This graph shows the efficiency curves which were measured for electrical motors constructed from core materials treated by different annealing methods. It is evident that the better efficiency (solid blue line) has the electrical motor which uses the segments treated in laboratory conditions with the high heating rate. The electrical motor, which was completely finished in conventional industrial conditions (dash orange line), exhibits lower efficiency within the whole range of torque load. It can be seen that the highest value of efficiency was obtained for the motor with laboratory (dynamically) heat treated segments in the range of the torque load from 0.15 Nm up to 0.38 Nm. The comparison of the obtained data clearly shows that the dynamic heat treatment of electric motor segments, which were shear cut from semi-finished electrical steels, improves not only their microstructure, texture and core losses (coercive force), but it also significantly increases the resulting efficiency of electrical motor. The experimental data show that our dynamic heat treatment method enables for a significant improvement of the magnetic properties of electric motor core laminations. Specifically, more than a 1.2% efficiency increase at the load of 0.28 Nm was achieved for electric motor with dynamically heat treated laminations, in comparison with the motor manufactured from laminations heat treated according to conventional industrial conditions. Thus, the results of current investigation may represent promising challenges for the end users of semi-finished electrical steels, not only in terms of possible improvements of their products technical parameters, but also as a potential decrease of production costs related to the final heat treatment.

## 4. Summary and Conclusions

In the presented paper the effects of the different heat treatment conditions on the evolution of average grain size, texture and magnetic loss components of semi-finished non-oriented electrical steel were studied. The experimental samples in the form of rotor and stator segments after the shear cutting were processed by conventional long-term and unconventional dynamic heat treatment technology. The obtained results have clearly shown that the annealing process with rapid heating promotes the evolution of selective growth of coarse-grains with enhanced intensity rotated cube texture. The main observations and conclusions can be summarized as follows:

- The rapid heating at dynamic annealing conditions of semi-finished NO silicon steels leads to a significant increase of average grain size of the obtained microstructure. The distinct evolution of coarse-grained microstructure is related to the strain-induced grain boundary migration mechanism under the influence of steep temperature gradient through the steel sheet cross-section.
- The unconventional dynamic heat treatment of the investigated electric motor core segments resulted in a stronger texture optimization tendency (i.e. the weakening or total absence the γ-fibre and forming strong rotated cube texture) than in the case of using conventional long-term annealing process.
- The improvement of the texture characteristics illustrated that the used dynamic heat treatment can optimize soft magnetic properties of the investigated semi-finished steel, namely its magnetic isotropy in combination with magnetic coercivity.
- The magnetic properties measured at 50 Hz frequency clearly showed that the evolved microstructures and textures of the segments heat treated by two different procedures are directly responsible for their final magnetic characteristics. The segments which were unconventionally heat treated at higher temperature with rapid heating were characterised by lower value of watt losses (4.33 W/kg), compared to those of the segments which were conventionally heat treated at lower temperature with much slower heating (5.45 W/kg).
- The measurement of efficiency of electric motors constructed from the segments heat treated under two different heat treatment conditions have clearly shown, that in comparison to the conventional heat treatment technology, the application of our unconventional dynamic heat treatment leads to a significant efficiency improvement by more than 1.2%.

## Figures and Tables

**Table 1:** The magnetic properties of chosen samples of investigated segments.

Sample Type	Watt losses in AC Magnetic Field P (W/kg)	Coercivity in AC Magnetic Field H_C_ (A/m)	Coercivity in DC Magnetic Field H_C_ (A/m)
Semi-finished state	13.8	320	230
Long-term annealing treatment	5.45	116	58
Dynamic annealing treatment	4.33	91	20
